# THE HEALTH CARE COSTS OF DEMENTIA FOR MEXICANS AND MEXICAN AMERICANS IN MÉXICO AND THE UNITED STATES

**DOI:** 10.1093/geroni/igad104.0124

**Published:** 2023-12-21

**Authors:** Mónika López Anuarbe, Angela Gutierrez

**Affiliations:** Connecticut College, New London, Connecticut, United States; Ohio University, Athens, Ohio, United States

## Abstract

Dementia prevalence and costs affect subpopulations differently, generating healthcare and aging disparities. Most research on healthcare costs associated with dementia has overlooked cross-national comparisons among Hispanics in general and Mexicans in particular. This study utilizes the 2012-2018 Mexican Health and Aging Study (MHAS) and Health and Retirement Study (HRS) to assess: 1) relationships between geographic context and healthcare costs (hospitalization, medical, outpatient, prescription, dental); and 2) whether dementia status moderates the geographic context-healthcare costs relationship. We hypothesized that persons with dementia incurred higher healthcare costs in both countries, and that residents in less populated areas had lower healthcare costs than their counterparts. We used multivariable logistic regressions and generalized linear models, accounting for personal, healthcare access, and utilization factors. Results suggest that in 2012 (MHAS n = 15,723), persons with dementia in México had average healthcare costs that were 60.3% greater compared to those without dementia. Individuals with dementia also had a higher probability (11.9% p = 0.040) of incurring any healthcare costs in less densely populated areas (under 15,000 persons). In contrast, Hispanics participating in the HRS during that period (n=15,567), 58% of whom were of Mexican origin, did not have higher total healthcare costs if diagnosed with dementia, but incurred larger expenses in rural areas compared to their urban counterparts (p< 0.001). These results underscore how geographical context varies across countries and how dementia status can leave some individuals more financially vulnerable than others, affecting their access to healthcare services and overall health.

